# Commentary: Motion sickness patches for PONV in thyroid surgery patients — methodological insights and clinical implications

**DOI:** 10.1097/JS9.0000000000003444

**Published:** 2025-09-08

**Authors:** Bing Chen, Zhengrong Zheng

**Affiliations:** a Department of Thyroid and Breast Surgery, Xiamen Humanity Hospital, Fujian Medical University, Xiamen, China; b Department of Thyroid and Breast Surgery, The Second Affiliated Hospital of Fujian Medical University, Quanzhou, China

*Dear Editor,*

We read with great interest the recent publication by Yu *et al*^[1]^ titled “*Effects of Motion Sickness Patch on Postoperative Nausea and Vomiting in Female Patients Undergoing Thyroid Surgery: A Randomized Clinical Trial*.” This well-conducted randomized controlled trial provides valuable evidence on the potential role of motion sickness patches in reducing postoperative nausea and vomiting (PONV) among female thyroid surgery patients. The rigorous design and clinically relevant findings are commendable.

The study showed that motion sickness patches, when used alongside dexamethasone and palonosetron, significantly reduced the incidence of PONV within 24 hours postoperatively. The randomized, double-blind methodology and standardized perioperative management enhanced internal validity. This innovative and accessible strategy could benefit high-risk surgical populations.

While fully recognizing these strengths, several issues merit further discussion in future research:

First, although enrollment was restricted to women aged 18–65 years, no subgroup analyses across different age brackets were reported. Physiological and hormonal differences between younger and older women may influence both PONV risk and responsiveness to the patch. Future studies could consider age-stratified analyses to clarify differential treatment effects.

Second, although baseline and intraoperative variables were well balanced, several perioperative factors were not explicitly addressed, including preoperative diet, anxiety, sleep quality, and menstrual cycle phase. These may influence gastric emptying and gastrointestinal function, thereby affecting PONV susceptibility. Future trials might benefit from incorporating such factors to reduce residual confounding.

Third, outcome assessment could be further refined. Although PONV incidence was reported at several time intervals, the study did not distinguish PONV subtypes or employ time-to-event analyses (e.g., Kaplan–Meier curves). Such analyses may help elucidate onset, duration of protection, and cumulative risk. Future studies could explore these approaches to enhance mechanistic insights.

Fourth, while randomization reduced bias, the main analysis relied primarily on *χ*^2^ tests. Incorporating multivariable logistic regression or survival models might help adjust for potential residual confounders such as intraoperative fluid balance or subtle differences in analgesic management, thereby improving robustness and external validity.

In conclusion, Yu *et al* provide valuable evidence supporting the use of motion sickness patches as an adjunctive strategy for PONV prevention in female thyroid surgery patients. Addressing the above issues in future trials will strengthen mechanistic understanding and optimize clinical translation. We sincerely appreciate the authors’ important contribution and look forward to further progress in this field.

## Data Availability

All data analyzed or generated during this study are included in the article.
